# Development and Validation of an Ultra-Performance Liquid Chromatography–Tandem Mass Spectrometry Method to Determine Maduramicin in Crayfish (*Procambarus clarkii*) and Evaluate Food Safety

**DOI:** 10.3390/foods10020301

**Published:** 2021-02-02

**Authors:** Xiuge Gao, Pei Teng, Lin Peng, Hui Ji, Yawei Qiu, Xiaoxiao Liu, Dawei Guo, Shanxiang Jiang

**Affiliations:** 1 Joint International Research Laboratory of Animal Health and Food Safety, College of Veterinary Medicine, Nanjing Agricultural University, 1 Weigang, Nanjing 210095, China; vetgao@njau.edu.cn (X.G.); 2015107005@njau.edu.cn (P.T.); 2016207003@njau.edu.cn (L.P.); jihui@njau.edu.cn (H.J.); qiuyawei@njau.edu.cn (Y.Q.); 2015107006@njau.edu.cn (X.L.); gdawei0123@njau.edu.cn (D.G.); 2 Laboratory of Veterinary Pharmacology and Toxicology, College of Veterinary Medicine, Nanjing Agricultural University, 1 Weigang, Nanjing 210095, China

**Keywords:** maduramicin, ionophore antibiotic, UPLC-MS/MS, veterinary drug residue, crayfish, Haff disease

## Abstract

Maduramicin (MAD) is widely introduced into aquatic environments and results in the contamination of fish products. Worryingly, the consumption of MAD-contaminated crayfish (*Procambarus clarkii*) may induce symptoms of Haff disease. In this study, to monitor this potential contamination and to understand the residue and elimination characteristics of MAD in edible tissues of crayfish, a sensitive and efficient ultra-performance liquid chromatography–tandem mass spectrometry method was developed, validated, and applied. After extraction with acetonitrile and purification by solid-phase extraction column, multiple-reaction monitoring mass spectrometry with positive ionization mode was used to determine MAD’s residues. The limits of detection and of quantification were 6 μg·kg^−1^ and 20 μg·kg^−1^, respectively. The fortified recoveries ranged from 74.2% to 110.4%, with relative standard deviation of 1.2% to 10.1%. Furthermore, MAD was completely eliminated after 3 and 5 days from abdominal muscle and hepatopancreas tissues of crayfish, respectively. The maximum residue limits (MRLs) of MAD respectively was 200 μg·kg^−1^ in muscle and 600 μg·kg^−1^ in the hepatopancreas, and its withdrawal time in both edible tissues was 25.8 °C·d. Collectively, the results of this study indicate the proposed method is an efficient tool to evaluate the public health risk associated with crayfish consumption.

## 1. Introduction

Veterinary antibiotics are used extensively to control infectious diseases and thereby promote the safety and growth of animals in the livestock/poultry industry worldwide. However, owing to the rapidly increasing demand for animal-derived protein foods, the use of veterinary antibiotics in meat production is reaching very high levels. Although China has partially banned the use of antibiotics as growth promoters in feed because of increasing microbial resistance to drugs, coccidiostats are still approved as feed additives for controlling coccidiosis and promoting chickens’ growth in China (Announcement No. 194, of the Ministry of Agriculture and Rural Affairs of the People’s Republic of China). Among the approved coccidiostats, ionophore polyether antibiotics (IPAs) are now one of the most extensively used globally [1]. However, because of poor absorption in the gut, more than 80% of IPAs are excreted in the feces, and IPAs are then primarily introduced into aquatic environments through the direct discharge of fecal wastes [2,3]. These IPAs are highly stable in animal feces and have strong potential for transport from manure to soils and surface waters [4].

IPAs are considered as emerging environmental pollutants because of their intensive use in the modern husbandry industry and are quickly becoming a problem of global concern [5,6]. The presence of IPAs in environmental surface waters, soils, and sediments has been reported from the USA [3], the EU [7,8], Argentina [6], Brazil [9], and China [10]. In addition, IPAs were detected in bottom-sediment samples at higher concentrations than those present in water from the same streams [11]. These antibiotic pollutants not only threaten the eco-environmental quality of waters but also pose a risk to humans with transport through the aquatic food chain.

Among the approved IPAs, maduramicin (MAD) is the most toxic drug, frequently inducing intoxication in domestic animals [12,13] and fowls [14,15] as well as humans [16]. Typical symptoms of MAD-induced intoxication of humans includes rhabdomyolysis and acute renal failure, which are symptoms highly similar to those caused by Haff disease within 24 h following the consumption of fishery products. Since first identified in 1924 in Europe, Haff disease has now been reported in China, USA, Japan, Brazil, Sweden, and Russia [17,18,19]. Fishery products such as buffalo fish, pomfret, and crayfish (Procambarus clarkii) can deliver toxins that are in the aquatic environment and trigger the onset of Haff disease [20,21,22]. In China and the USA from 2009 to 2018, Haff disease case clusters appeared following the ingestion of cooked crayfish [18,22,23,24,25,26]. These outbreaks highlight the risks of crayfish consumption to the public, because eating crayfish is undoubtedly strongly linked to Haff disease. Although the toxin responsible for Haff disease remains unknown, a new heat-stable, algal toxin similar to palytoxin is suspected.

With the number of Haff disease cases associated with crayfish consumption increasing globally [25], it is urgent to identify the direct cause to correctly diagnose and control the disease. Therefore, the relation between MAD in aquatic environments and crayfish consumption-associated Haff disease needs to be investigated. However, to explore the relation, a detection method for MAD in crayfish must be developed. Crayfish are very important as components of aquatic ecosystems and also from a human health perspective. Because crayfish tolerate polluted aquatic environments; they are suitable bioindicators of toxic pollutants [27,28]. Moreover, crayfish consumption can be a key route by which environmental antibiotics and heavy metals are transferred into humans [29]. Therefore, to obtain information on this exposure route, monitoring MAD in aquatic environments is required.

Considering the residue and potential risk of MAD to humans and environment, numerous methods for the analytical determination of MAD and other ionophores in biological matrices (e.g., feed (LC–MS/MS), eggs (HILIC–MS/MS and electrochemical immunosensor), liver (LC–MS/MS), human plasma (LC–MS/MS), poultry tissue (LC–MS/MS)) or environmental matrices (e.g., surface water and sediment (HPLC–MS, LC–MS/MS)) have been widely reported in the literatures [2,11,30,31,32,33,34,35]. To our knowledge, However, a method is not currently available to reliably detect MAD in crayfish.

Because of the emergence of Haff disease caused by crayfish consumption, likely associated with the extensive use of MAD in livestock production and its increasing frequency in aquatic environments, it is urgent to develop a detection method in order to understand the relations between MAD and crayfish-related Haff disease. In this study, a sensitive and reliable method was developed to determine MAD residues in edible tissues of crayfish. The method used a cleanup by solid-phase extraction (SPE) coupled with ultra-performance liquid chromatography–tandem mass spectrometry (UPLC–MS/MS) with electrospray positive ionization (ESI+) under the multiple-reaction monitoring (MRM) mode. With the method, the residue and elimination characteristics of MAD in crayfish were investigated, in addition to determining the maximum residue limits (MRLs) and withdrawal time (WT).

## 2. Materials and Methods

### 2.1. Reagents and Materials

Maduramicin ammonium (maduramicin, MAD, C_47_H_83_NO_17_, 934.17 MW, purity > 92.3%) was used as the standard compound, purchased from the China Institute of Veterinary Drug Control (Beijing, China). Nigericin sodium (nigericin—NIG, C_40_H_67_NaO_11_, 746.94 MW, purity > 98.0%) was used as the internal standard, was supplied by MedChemExpress (Monmouth, US). The Esigmabio Co., Ltd. (Haining, Zhejiang, China) kindly donated the maduramicin (purity > 91.9%, batch number 1701004) used to test crayfish exposure.

Deionized water was taken from the Milli-Q water purification system (Millipore, France). Methanol (MeOH) and acetonitrile (ACN) were of LC-MS grade and purchased from Merck (Darmstadt, Germany). Analytical purity grade citric acid monohydrate, trisodium citrate dehydrate, anhydrous glucose, formic acid, n-hexane, sodium diacetate, and sodium sulfate anhydrous, all of analytical purity grade, were bought from Aladdin (Shanghai, China). The Oasis HLB solid-phase extraction (SPE) column was obtained from Waters (60 mg, 3 mL; Milford, MA, USA). Blank crayfish used to analyze the specificity of the UPLC-MS/MS method were purchased from local markets (Suguo supermarket in Nanjing, China). These crayfish were acclimatized for seven days for seven days before investigating the selectivity of the LC–MS/MS method to determine MAD.

### 2.2. Experimental Animals and Sample Pre-Preparation

Adult crayfish (weight, 21.65 ± 1.26 g) were purchased from the Freshwater Fisheries Research Institute of Jiangsu Province (Nanjing, Jiangsu, China). All obtained crayfish were antibiotic-free and in good health. These crayfish were acclimated to dechlorinated tap water (20 ± 2°C) with continuous aeration in plastic aquaria (730 mm × 560 mm × 230 mm) under laboratory conditions for seven days (using a photoperiod of 14-h light/10-h dark). They were fed once a day with commercial pellets free of any antibiotics or drugs. To avoid cannibalism of crayfish, several polyvinyl chloride (PVC) tubes were placed in aquaria as shelters. Water was replaced every 48 h to ensure a high-quality environment for crayfish culture. Negative control tissue samples to establish the method were blank hepatopancreas and abdominal muscle tissues (i.e., without the intestine cord) dissected and stored at −80°C.

### 2.3. Standard Solutions

Stock solutions containing 1 mg·mL^−1^ MAD or 4 mg·mL^−1^ NIG were prepared by dissolving standard chemicals in acetonitrile and then stored at −20°C, in the dark. Working solutions of MAD and NIG spanning a suitable range were obtained through a gradient dilution of the stock solutions in acetonitrile and then stored at 4 °C in the dark.

### 2.4. Ultra-Performance Liquid Chromatography–Tandem Mass Spectrometry Conditions

Ultra-performance liquid chromatography–tandem mass spectrometry was performed on a Waters Acquity UPLC system (Waters, Milford, MA, USA) coupled to a Xevo TQD triple quadrupole mass spectrometer (Waters, Milford, MA, USA) equipped with an electrospray ion source. The UPLC–MS/MS instrument was controlled by the software workstation of MassLynx 4.1 (Waters, Milford, MA, USA).

#### 2.4.1. Chromatography

An Acquity UPLC BEH C18 column (50 mm × 2.1 mm, particle size = 1.7 μm; Waters, Milford, MA, USA) was used an oven temperature of 30 °C. The mobile phase consisted of 10% elution A (ultrapure water that contained 0.1% formic acid) and 90% elution B (acetonitrile with 0.1% formic acid), at a flow rate 0.35 mL·min^−1^. The injected sample volume was 1 μL.

#### 2.4.2. Mass Spectrometry (MS)

To quantify MAD and NIG, tandem MS was conducted under the MRM mode and in the ESI+ mode. The optimized parameters were as follows: capillary voltage, 3.0 kV, ionization source temperature, 150 °C, desolvation temperature, 350 °C with a flow rate of 600 L·h^−1^; cone gas flow rate, 50 L·h^−1^. Mass spectrometric parameters of MAD and NIG are provided in Supplemental Table S1. The MS parameters were optimized by injection of individual standard solutions of MAD (200 ng·mL^−1^) and NIG (200 ng·mL^−1^). Analyte identification was based on the relative retention time and the relative percent ion ratio of the qualitative ion/quantitative ion.

### 2.5. Sample Preparation

Edible tissues of crayfish (i.e., hepatopancreas and abdominal muscle) were weighed (1.00 ± 0.01 g) into 50-mL polypropylene screw-cap tubes and spiked with a 100-μL internal standard working solution (4 µg·mL^−1^). After standing for 10 min, 3 mL of acetonitrile was added to each sample. Samples were then homogenized for 30 s using an Ultra-Turrax homogenizer (IKA, Staufen, Germany). The homogenizer shearing head was washed using 2 mL of acetonitrile to reduce the loss of standard working solution. Furthermore, the wash was combined with the homogenate in the same 50-mL tube. Anhydrous sodium sulfate (1 g) was spiked into each tested tube, followed by vertical vortexing for 3 min, ultrasonic extraction for 10 min, and centrifugation for 5 min at 3387 g at 4 °C. The ensuing supernatant was transferred into a clean 50-mL centrifuge tube. Then, the residue was re-dissolved by adding 5 mL of acetonitrile and extracted twice, and the supernatants were collected and mixed with the previously obtained extract. After the addition of 5 mL of acetonitrile (containing saturated N-hexane) into each tube, the mixtures were shaken on a vortex mixer for 2 min. After standing to allow for stratification, the N-hexane layer was removed. The extracts were evaporated to dryness under N_2_ stream at 50 °C. The residues were re-dissolved in 10 mL of a 50% methanol solution containing 0.5% sodium acetate by weight.

In a comparison of acetonitrile, methanol, dichloromethane, and isooctane for use in the MAD extraction phase, acetonitrile was selected as the optimum extracting-solvent because of its good recovery and low matrix effects. The choice of acetonitrile is consistent with a recent report that detected multiple pharmaceuticals in crayfish [28]. Furthermore, in a comparison of the SPE Oasis HLB, Strata C18, and Sep-Pak Silica columns for use in removing other impurities in the samples before loading them into the UPLC–MS/MS system, the Oasis HLB columns had the highest purification efficiency and recovery for the prepared MAD samples of crayfish tissues (Supplementary Materials Table S2). In the next purification step, the re-dissolved supernatant was purified via a solid-phase extraction (SPE) column (Oasis HLB, 60 mg, 3 mL; Waters, Milford, MA, USA). The SPE column was activated with 3 mL of methanol and then 3 mL of deionized water. First, 2 mL of re-dissolved supernatant was passed through the activated SPE column under the action of gravity; then the column was washed using 3 mL of deionized water. Next, the column was dried, using a vacuum pump. Last, the residues were eluted from the SPE column with 3 mL of methanol, and the eluent was dried under N_2_ at 50 °C. Concentrated residues were reconstituted in 2 mL of the mobile phase and filtered through 0.22-μm syringe filters before subjecting them to UPLC-MS/MS.

### 2.6. Validation of The Method

The in-house validation of the proposed method was based on accepted criteria for the quality control of laboratory chemical testing of food (GB/T 27404-2008) and for the quality control of laboratories for the residue analysis of veterinary drugs (NY/T 1896–2010), including specificity investigation, calibration curve, recovery (precision and accuracy), limit of detection (LOD), limit of quantification (LOQ), stability, and matrix effects (MEs).

#### 2.6.1. Selectivity Investigation

To understand the selectivity of the proposed method, more than 10 blank samples of crayfish obtained from several local supermarkets were analyzed. The chromatograms of blank hepatopancreas and abdominal muscle tissues of crayfish were compared against the chromatograms of standard solutions (1 ng·mL^−1^ MAD and 20 ng·mL^−1^ NIG) in a pure solvent and blank tissues spiked with the target analytes (20 μg·kg^−1^ MAD and 400 μg·kg^−1^ NIG). The aim of this process was to avoid obtaining a false positive signal or a false negative signal.

#### 2.6.2. Linearity Range and Matrix Effects (ME)

Linearity was assessed in both standard working solutions, and also in the spiked standard samples, which were obtained by spiking standards into the extract after sample extraction. To construct a standard solution curve and matrix-matched calibration curves, standard solutions of MAD (20, 100, 200, 400, 1000, 2000, 4000 and 8000 µg·L^−1^) and NIG (400 µg·L^−1^) in the mobile phase were prepared. The final fortified MAD at eight concentrations (20, 100, 200, 400, 1000, 2000, 4000 and 8000 μg·kg^−1^) in crayfish hepatopancreas and abdominal muscle tissues was analyzed by the proposed UPLC-MS/MS method. The relative peak areas of analytes were plotted versus known concentrations, and the regression equations and their coefficients of determination (r) were calculated. The MEs were investigated by analyzing the slopes of standard curves drawn between solvent-only and matrix-matched standard samples. The ME was calculated as follows: ME (%) =B/A × 100, where A is the slope ratio of calibration curves with standards prepared with solvent and B is the slope ratio of matrix-matched calibration curves.

#### 2.6.3. Recovery and Precision

The rate of recovery was obtained by calculating the percentage of measured drug concentration versus the fortified drug concentration. Blank samples that received an addition of MAD (20, 200, 1000, or 4000 μg·kg^−1^) and NIG (400 μg·kg^−1^) before extraction were analyzed on three consecutive days (*n* = 6 for each concentration). Accuracy and precision were evaluated on the basis of intra- and inter-day repeatability, presented here as relative standard deviations (RSDs [%]).

#### 2.6.4. Limits of Detection (LOD) and Quantification (LOQ)

The LOD and LOQ of the proposed method were calculated by measuring the ratio of signal to noise (S/N) at the retention time of a given target analyte. In this study, an S/N ≥ 3 was used for LOD and an S/N ≥ 10 was used for LOQ.

#### 2.6.5. Sample Stability

To understand the stability of samples with MAD and ensure reliable data were obtained, three storage methods for blank hepatopancreas and muscle tissues with a range of MAD concentrations (20, 200, 4000 μg·kg^−1^) were investigated. Stability was investigated with storage at room temperature for 6 h (short-term) and at −80 °C for 30 d (long-term). The freeze–thaw stability was investigated in three cycles from −80 °C to room temperature on three consecutive days. All samples were determined according to the above-mentioned procedure and analyzed by UPLC-MS/MS. The recoveries and RSD (%) of the tested samples were then calculated.

### 2.7. Applications in Crayfish Samples Exposed to MAD

**Figure 1:**
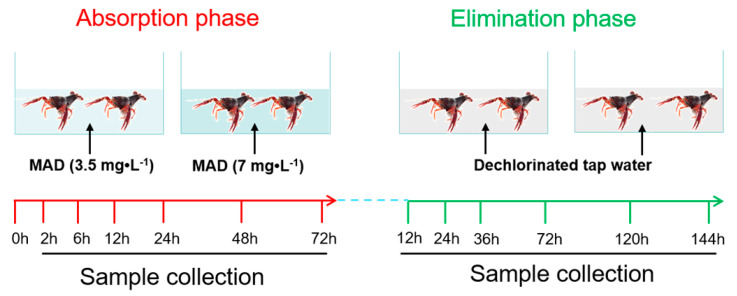
Experimental design for the absorption and elimination phases of maduramicin (MAD) in crayfish.

To investigate the absorption and elimination characteristics of MAD in edible tissues of crayfish, an exposure study was conducted using a two-stage process in a semi-static manner. A total of 432 male crayfish were randomly divided into two groups, in triplicate, which were treated respectively with either 7 mg·L^−1^ (1/10 the LC_50_) or 3.5 mg·L^−1^ (1/20 the LC_50_) of MAD (Figure 1). Their culture conditions were the same as those described in the acclimation period. In the absorption phase, crayfish were exposed to MAD for 72 h. The duration was determined in a pre-study in which crayfish were exposed to MAD from 24 h to 120 h, and MAD levels in hepatopancreas and abdominal muscle tissues reached a steady state in 72 h. During the exposure period, water with MAD was changed daily to maintain the MAD concentration. After 2, 6, 12, 24, 48, and 72 h of exposure, the hepatopancreas and abdominal muscle of six crayfish per group were obtained and stored at –80°C. At these same time points during the MAD exposure, water samples were also collected. The concentration of MAD in water was determined by using a previously established UPLC-MS/MS method of our group [36]. This analysis demonstrated that a relatively stable concentration was maintained throughout the absorption phase (Supplementary Materials Figure S1).

In the elimination phase, the remaining crayfish of each group were transferred into freshwater without MAD for a total of 144 h. During this period, the water was changed daily. The hepatopancreas and abdominal muscle of six crayfish in each group were collected after 12, 24, 36, 72, 120, and 144 h to determine the elimination of MAD. Samples were stored at −80°C prior to their UPLC-MS/MS analysis.

### 2.8. Maximum Residue Limits (MRLs) and Withdrawal Time (WT)

On the basis of the MAD residue data, the MRLs in edible crayfish tissues were established according to the Codex Alimentarius Commission. The MRLs were calculated as follows:

$$\mathrm{MRLs}=\frac{\mathrm{ADI}\times \mathrm{Human}\;\mathrm{weight}}{\mathrm{Daily}\;\mathrm{food}\;\mathrm{intake}}$$

where ADI is the abbreviation for acceptable daily intake, set to 1 μg·kg^-1^ body weight for MAD as per the Scientific Committee on Animal Nutrition (European Food Safety Authority, 2008), and human weight was fixed at 60 kg. For hepatopancreas and muscle tissues, the daily food intake was 0.1 kg/day and 0.3 kg/day, respectively.

In addition, to calculate the WT of MAD in the hepatopancreas and abdominal muscles of crayfish, the data on concentration vs. time from the elimination phase were used in a statistical software program, WT1.4, adopted by the European Medicines Agency (EMA). The WT values were determined as those times when the concentrations of MAD in the crayfish hepatopancreas or abdominal muscles were below the MRL within a given confidence level (95%). Because the pharmacokinetic dynamics of drugs in fish are altered by water temperature, WT was expressed here as “°C·d” by multiplying the mean daily water temperature (°C) tested during the entire experimental period.

### 2.9. Data Analyses

Data were summarized in MS Excel. The MAD absorption and elimination data in crayfish are expressed as the mean ± SD (standard deviation). Statistical analysis in the experiments was performed using the SPSS 18.0.

## 3. Results and Discussion

### 3.1. Chromatographic Separation and Mass Spectrometric Optimization

**Figure 2:**
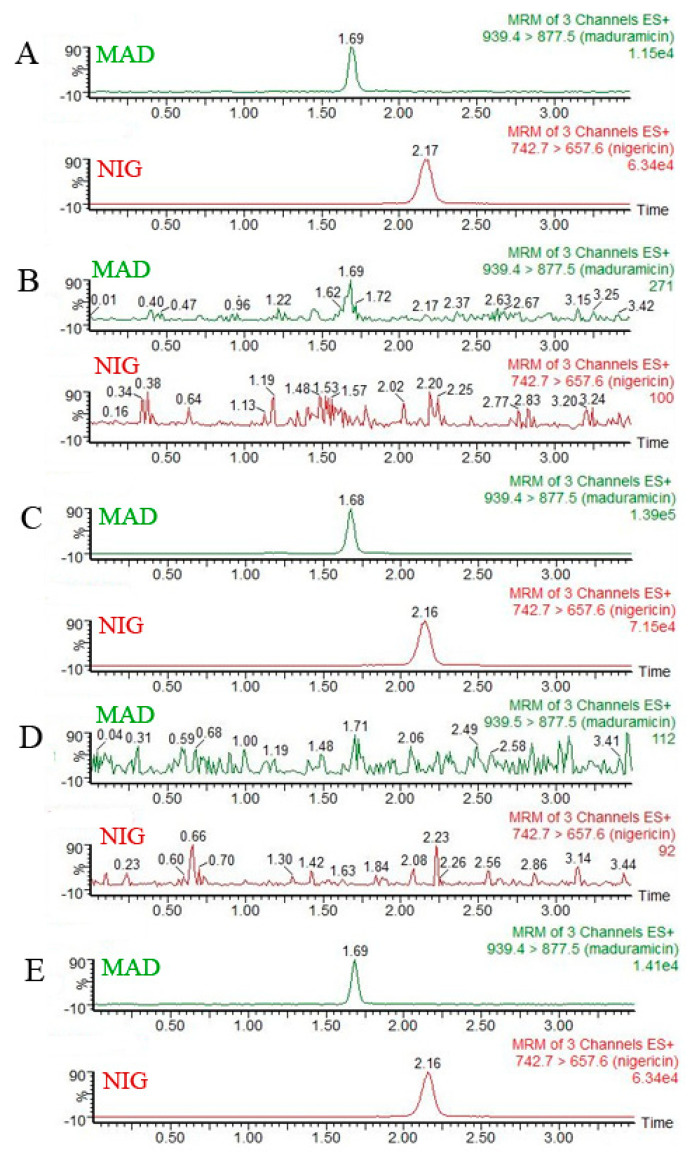
Mass chromatograms of standard maduramicin (MAD) and nigericin (NIG) solutions, blank tissues, and blank tissues spiked with MAD and NIG. Quantitative ion mass spectra of MAD and NIG: (**A**) 1 ng·mL^−1^ standard MAD solution; 20 ng·mL^−1^ standard NIG solution. (**B**) Blank hepatopancreas tissues of crayfish. (**C**) 20 µg·kg^−1^ MAD and 400 µg·kg^−1^ NIG spiked in the hepatopancreas of crayfish. (**D**) Blank abdominal muscle of crayfish. (**E**) 20 µg·kg^−1^ MAD and 400 µg·kg^−1^ NIG spiked in the abdominal muscle of crayfish.

The mobile phase, chromatographic column, and sample volume were selected to optimize UPLC conditions. Four commonly used mobile phases were tested in a pre-experiment: methanol and water, acetonitrile and water, methanol and formic acid, acetonitrile and formic acid. Acetonitrile was selected as the most suitable solvent because it provided good baseline separation, symmetrical peak shape, and a short retention time of MAD (data not shown). The analysis time of the two target compounds was ca. 3.5 min (Figure 2). To obtain greater separation efficiency of MAD, the Acquity UPLC BEH C18 column was selected. The addition of 0.1% formic acid in the aqueous phase improved the MAD ion response signal, a result consistent with a previously reported UPLC–MS/MS method to determine drug residues in crayfish tissues [28]. The most suitable injection volume of MAD was identified to be 1 μL. In the quantification of MAD in food samples, nigericin is often selected as the internal standard substance [31]. In this study, the performance of nigericin was validated in the analysis of crayfish tissues.

### 3.2. Specificity and Sensitivity

Firstly, to explore the specificity of the proposed method, more than 10 individual, control crayfish samples from different sources were tested. These results indicated there was no interference in hepatopancreas and abdominal muscle tissues affecting the retention time of MAD and NIG (data not shown). Additionally, the LOD and LOQ in these two crayfish tissues were 6 μg·kg^−1^ and 20 μg·kg^−1^, respectively. These values are higher than those found in chicken meat (LOD= 0.08 ng g^−1^, LOQ = 0.28 ng g^−1^) [32] and sediment samples (LOQ= 1 µg/kg) [11], which were determined by using different instrumentation with a higher-level configuration. Nevertheless, the values are compatible with the LOQs of compounds analyzed in crayfish samples at the μg·kg^−1^ level [28].

### 3.3. Linearity and Matrix Effects (ME)

The standard curve obtained from the standard MAD working solutions (20, 100, 200, 400, 1000, 2000, 4000, and 8000 ng·mL^−1^) and the calibration curve from blank crayfish tissues spiked with MAD (20, 100, 200, 400, 1000, 2000, 4000, and 8000 μg·kg^−1^) showed good linearity, with R^2^ > 0.99 for all tested samples. The regression equations and R^2^ values of MAD in the spiked hepatopancreas and abdominal muscle are presented in Supplementary Materials Table S3.

**Figure 3:**
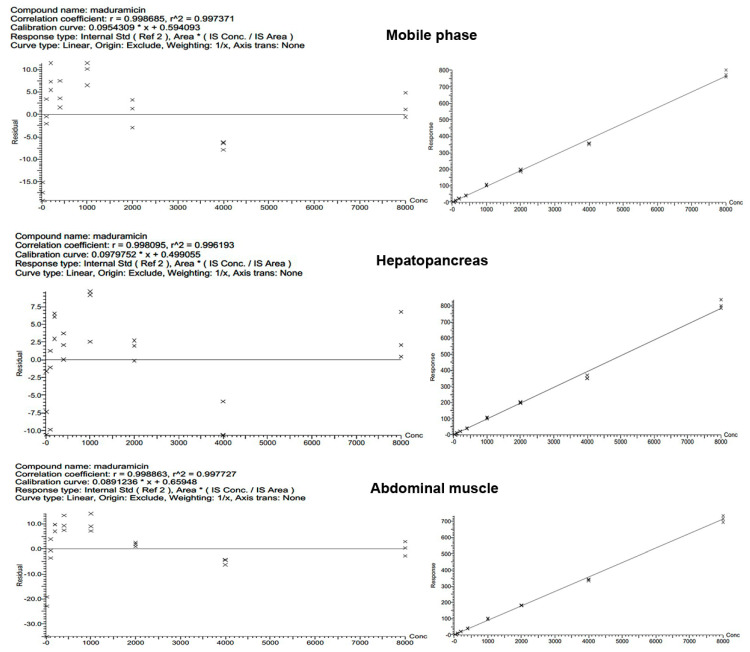
Matrix effects of crayfish hepatopancreas and muscle tissues on the analysis of maduramicin.

To assess MEs, the slopes of mobile phase-only and the matrix-matched MAD standards were compared at the same concentrations. The calibration curve slopes of solvent-only, hepatopancreas, and muscle were 0.095, 0.098, and 0.089, respectively (Figure 3). According to the derived slope of the matrix-matched calibration curve, the MEs of hepatopancreas and muscle were 103.2% and 93.7%, respectively, which fell within the range of 0.8 to 1.2 [37], indicating that the MEs could be ignored [38], a finding consistent with a recent study [39]. Next, the standard solution curve was used to quantify the MAD residue in this study. However, in the multi-residue analysis of feedstuff or animal-derived food samples, matrix-matched calibration curves are commonly used in the quantification of target compounds [31,34].

### 3.4. Accuracy and Precision

MAD was spiked at four levels in the two edible tissues of crayfish, with every spiked sample determined with six replicates (Table 1). The recovery rates of MAD at all levels ranged between 82.3% and 92.4% in the hepatopancreas and between 78.5% and 110.4% in crayfish muscle. The inter- and intra-level relative standard deviations (RSDs [%]) of MAD were <15% for all detected samples and different levels of MAD, suggesting the method had reliable within-laboratory reproducibility according to the accepted criteria for the quality control of laboratory chemical testing of food (GB/T 27404-2008) and criteria on quality control of laboratories for the residue analysis of veterinary drugs (NY/T 1896–2010).

### 3.5. Sample Stability

The stability of samples during preparation and UPLC-MS/MS was investigated by spiking with MAD at three levels in the tested two tissues. The stability of MAD stock solution was analyzed in a preliminary test, and MAD was stable for at least 30 days in stock solution at −20°C. Throughout the test period, the recovery of MAD at the three levels under the three storage conditions was between 73.3% and 108.8% (Supplementary Materials Table S4). Thus, the stability of MAD in crayfish hepatopancreas and muscle tissues was good, making them suitable for subsequent analyses.

### 3.6. Absorption and Elimination of MAD in Crayfish Hepatopancreas and Muscle Tissues

To explore the residue and elimination characteristics of MAD, crayfish were exposed to a solution spiked with MAD (low level: 3.5 mg·L^−1^ vs high level: 7 mg·L^−1^) in a 72-h absorption phase, followed by a 6-d elimination phase without MAD exposure, based on the findings of preliminary experiments (data not shown). In crayfish hepatopancreas and muscle tissues, MAD rapidly reached a steady-state level after 72 h of exposure (Table 2). With an increase in time of exposure in the absorption phase, the MAD residue levels in the two tissues increased in concentration- and time-dependent manners. However, in the elimination phase, the elimination rate of MAD in muscle was faster than that in the hepatopancreas, with MAD residue undetectable on day three (low level) and day five (high level) in muscle tissues. On day 6, MAD (low and high levels) was also undetectable in the hepatopancreas. Thus, crayfish hepatopancreas tissue containing MAD likely poses a greater risk to consumers than contaminated abdominal muscle, and this information should be made known to the general public. The residue characteristics of MAD in the edible tissues of crayfish are similar to those in broiler chickens, which have the highest concentrations of MAD in the heart (285.8 ng·g^−1^) followed by those in the liver (146.8 ng·g^−1^) after withdrawal on day 0 [30]. In addition, according to Chang et al., also on day 0 after withdrawal, MAD residues in liver and breast are 627.0 ± 9.6 ng·g^−1^ and 52.9 ± 5.4 ng·g^−1^, respectively [32]. Both studies show rapid absorption and elimination rates of MAD in chickens [30,32]. These results further indicate the proposed UPLC–MS/MS method successfully determined the presence and amounts of MAD in edible crayfish tissues.

### 3.7. Maxiumum Residue Limits and Withdrawal Times of MAD in Crayfish

The MRLs of MAD in food derived from broiler chickens have been established in most countries, including China, the EU, the US, and Japan. However, for aquatic products, to our best knowledge, there are currently no available MRLs for MAD anywhere in the world. To better protect the health of consumers, MRLs should be established in accordance with generally recognized principles of safety assessment from the Codex Alimentarius Commission, taking into account toxicological risks and environmental contamination, as well as the pharmacological effects of residues. According to the European Food Safety Authority published ADI value (= 1 μg·kg^−1^ body weight) of MAD for humans, the MRLs of MAD in crayfish tissues in this study were calculated as 200 μg·kg^-1^ in abdominal muscle and 600 μg·kg^−1^ in hepatopancreas. The WTs of MAD at all tested levels and samples are shown in Table 3. In the hepatopancreas with low and high levels of MAD exposure, the respective WTs were 4.6 °C·d and 25.8 °C·d. However, WTs could not be calculated for muscle tissue because MAD residues were lower than the MRL. These findings thus provide useful information to avoid MAD contamination-induced harm to human health.

## 4. Conclusions

In summary, the proposed UPLC-MS/MS method is a reliable way to determine MAD in the edible tissues of crayfish (*P. clarkii*). Notably, the extraction and cleanup procedures were optimized to obtain a high recovery of MAD and to yield results quickly. Validation results showed the proposed method provided good linearity, sensitivity, repeatability, acceptable stability, and negligible matrix effects, thus providing a valid and robust method well suited for the purpose of detecting MAD at trace levels in crayfish. Thus, the method was valid and robust and well suited to detect MAD at trace levels in crayfish. The method successfully analyzed the residue and elimination characteristics of MAD in crayfish, determining that MAD was undetectable in abdominal muscle and hepatopancreas tissues after three and six days of elimination, respectively. To help safeguard human health, the MRLs of MAD in crayfish abdominal muscle (200 μg·kg^−1^) and hepatopancreas (600 μg·kg^−1^) tissues were determined for the first time, in addition to its WT (25.8 °C·d). The method is amenable to quantitative analysis and for the confirmation of MAD in foods of crayfish and should prove useful in reducing the risk to personal health due to consumption of MAD-contaminated crayfish.

## Figures and Tables

**Table 1:** Recovery and precision of the ultra-performance liquid chromatography–tandem mass spectrometry method to determine maduramicin in edible crayfish tissues (*n* = 6 at each tested concentration, on three consecutive days).

Sample	Spiked Concentration (μg·kg^-1^)	Mean Recovery (%)	Inter-RSD (%)	Intra-RSD (%)
Hepatopancreas	20	83.8	8.0	8.1
	200	89.1	2.4	3.7
	1000	90.5	2.0	2.9
	4000	87.5	2.2	4.5
Abdominal muscle	20	82.2	3.7	5.0
	200	108.0	1.4	1.9
	1000	106.7	2.0	2.7
	4000	101.3	3.1	3.2

Note: RSD is the relative standard deviation percentage (%).

**Table 2:** Residue and elimination characteristics of maduramicin (MAD) in abdominal muscle and hepatopancreas tissues of crayfish (*n* = 6).

Treatment Time (h)		MAD in Muscle (μg·kg^−1^)		MAD in Hepatopancreas (μg·kg^−1^)	
		Low	High	Low	High
Absorption phase	2	35.22 ± 10.71	47.13 ± 11.89	87.65 ± 30.52	110.96 ± 73.00
	6	40.41 ± 5.31	58.13 ± 10.26	107.73 ± 31.92	207.89 ± 48.36
	12	56.13 ± 22.85	65.25 ± 10.58	185.99 ± 50.17	362.86 ± 72.63
	24	49.47 ± 6.77	62.49 ± 9.71	280.89 ± 101.45	541.56 ± 158.18
	48	69.75 ± 23.44	115.45 ± 18.09	369.10 ± 101.09	672.21 ± 134.29
	72	75.63 ± 7.32	105.12 ± 37.01	364.90 ± 49.31	689.55 ± 162.84
Elimination phase	12	42.24 ± 19.48	51.80 ± 17.85	150.90 ± 59.66	224.04 ± 80.48
	24	33.57 ± 17.27	47.83 ± 11.32	135.20 ± 44.72	245.01 ± 146.22
	36	29.31 ± 9.66	46.58 ± 10.14	73.17 ± 24.25	152.05 ± 51.54
	72	ND	20.80 ± 0.54	30.12 ± 8.40	68.29 ± 32.94
	120	ND	ND	28.89 ± 5.33	31.91 ± 7.39
	144	ND	ND	ND	ND

Note: ND indicates no detection, suggesting the residue concentration of MAD was lower than the limit of detection (LOD).

**Table 3:** Maximum residue limits (MRLs) and withdrawal times (WTs) of two levels of maduramicin (MAD) in crayfish.

Group	Sample	MRLs (μg·kg^−1^)	WT (°C·d)
Low MAD (3.5 mg·L^−^^1^)	Hepatopancreas	600	4.6
	Abdominal muscle	200	--
High MAD (7 mg·L^−^^1^)	Hepatopancreas	600	25.8
	Abdominal muscle	200	--

Note: -- indicates WT could not be determined.

## Supplementary Materials

The following are available online at https://www.mdpi.com/2304-8158/10/2/301/s1, Table S1: Mass spectrometric parameters of MAD and NIG, Figure S1: Concentration of MAD in water sample, Table S2: Recovery rates of MAD in hepatopancreas and abdominal muscle of crayfish using three solid phase extraction columns (*n* = 3), Table S3: Standard solution curve and matrix-matched calibration curves of MAD, Table S4: Stability test results of MAD under three storage conditions (*n* = 3).

## References

[1] Noack S., Chapman H.D., Selzer P.M. (2019). Anticoccidial drugs of the livestock industry. Parasitol. Res..

[2] Sun P., Barmaz D., Cabrera M.L., Pavlostathis S.G., Huang C.H. (2013). Detection and quantification of ionophore antibiotics in runoff, soil and poultry litter. J. Chromatogr. A.

[3] Cha J., Carlson K.H. (2018). Occurrence of beta-lactam and polyether ionophore antibiotics in lagoon water and animal manure. Sci. Total Environ..

[4] Biswas S., McGrath J.M., Sapkota A. (2012). Quantification of ionophores in aged poultry litter using liquid chromatography tandem mass spectrometry. J. Environ. Sci. Health B.

[5] Hansen M., Krogh K.A., Björklund E., Halling-Sørensen B., Brandt A. (2009). Environmental risk assessment of ionophores. TrAC Trends Anal. Chem..

[6] Alonso L.L., Demetrio P.M., Capparelli A.L., Marino D.J.G. (2019). Behavior of ionophore antibiotics in aquatic environments in Argentina: The distribution on different scales in water courses and the role of wetlands in depuration. Environ. Int..

[7] Herrero P., Borrull F., Marce R.M., Pocurull E. (2013). Determination of polyether ionophores in urban sewage sludge by pressurised liquid extraction and liquid chromatography-tandem mass spectrometry: Study of different clean-up strategies. J. Chromatogr. A.

[8] Bak S.A., Björklund E. (2014). Occurrence of Ionophores in the Danish Environment. Antibiotics.

[9] Yopasa-Arenas A., Fostier A.H. (2018). Exposure of Brazilian soil and groundwater to pollution by coccidiostats and antimicrobial agents used as growth promoters. Sci. Total Environ..

[10] Zhou L.J., Wang W.X., Lv Y.J., Mao Z.G., Chen C., Wu Q.L. (2020). Tissue concentrations, trophic transfer and human risks of antibiotics in freshwater food web in Lake Taihu, China. Ecotoxicol. Environ. Saf..

[11] Kim S.C., Carlson K. (2006). Occurrence of ionophore antibiotics in water and sediments of a mixed-landscape watershed. Water Res..

[12] Dorne J.L., Fernandez-Cruz M.L., Bertelsen U., Renshaw D.W., Peltonen K., Anadon A., Feil A., Sanders P., Wester P., Fink-Gremmels J. (2013). Risk assessment of coccidostatics during feed cross-contamination: Animal and human health aspects. Toxicol. Appl. Pharmacol..

[13] Shimshoni J.A., Britzi M., Pozzi P.S., Edery N., Berkowitz A., Bouznach A., Cuneah O., Soback S., Bellaiche M., Younis A. (2014). Acute maduramicin toxicosis in pregnant gilts. Food Chem. Toxicol..

[14] Gao X., Peng L., Ruan X., Chen X., Ji H., Ma J., Ni H., Jiang S., Guo D. (2018). Transcriptome profile analysis reveals cardiotoxicity of maduramicin in primary chicken myocardial cells. Arch. Toxicol..

[15] Gao X., Zheng Y., Peng L., Ruan X., Ji H., Qiu Y., Liu X., Teng P., Guo D., Jiang S. (2018). Maduramicin induces apoptosis in chicken myocardial cells via intrinsic and extrinsic pathways. Toxicol. In Vitro.

[16] Sharma N., Bhalla A., Varma S., Jain S., Singh S. (2005). Toxicity of maduramicin. Emerg. Med. J..

[17] Buchholz U., Mouzin E., Dickey R., Moolenaar R., Sass N., Mascola L. (2000). Haff disease: From the Baltic Sea to the U.S. shore. Emerg. Infect. Dis..

[18] Diaz J.H. (2015). Global incidence of rhabdomyolysis after cooked seafood consumption (Haff disease). Clin. Toxicol..

[19] Chan T.Y. (2016). The emergence and epidemiology of Haff disease in China. Toxins.

[20] Herman L.L., Bies C. (2014). Haff disease: Rhabdomyolysis after eating buffalo fish. West. J. Emerg. Med..

[21] He F., Ni J., Huang J.A., Liu Y., Wu C., Wang J. (2018). Clinical features of Haff disease and myositis after the consumption of boiled brackish water crayfish: A retrospective study of 96 cases at a single centre. Intern. Emerg. Med..

[22] Huang C., Peng L., Gong N., Xue C., Wang W., Jiang J. (2019). A retrospective analysis of crayfish-related rhabdomyolysis (Haff disease). Emerg. Med. Int..

[23] Zhang B., Yang G., Yu X., Mao H., Xing C., Liu J. (2012). Haff disease after eating crayfish in east China. Intern. Med..

[24] Bai L., Xu M.J., Li W.W., Han H.H., Liu J.K., Fu P., Xu L.Z., Ouyang Y.Y., You X.Y., Chen J. (2019). Retrospective case analysis of crayfish-transmitted Haff disease in China during 2016–2017. Food Control.

[25] Pei P., Li X.Y., Lu S.S., Liu Z., Wang R., Lu X.C., Lu K. (2019). The emergence, epidemiology, and etiology of Haff disease. Biomed. Environ. Sci..

[26] Chen Y., Yuan B.J., Xie G.X., Zhen S.Q., Zhou Y.J., Shao B., Zhang J., Ji H., Wu Y.N. (2016). Outbreak of Haff Disease caused by consumption of crayfish (*Procambarus clarkii*), Nanjing, Jiangsu Province, China. Food Control.

[27] Schilderman P.A.E.L., Moonen E.J.C., Maas L.M., Welle I., Kleinjans J.C.S. (1999). Use of crayfish in biomonitoring studies of environmental pollution of the river Meuse. Ecotoxicol. Environ. Safe..

[28] Kazakova J., Fernandez-Torres R., Ramos-Payan M., Bello-Lopez M.A. (2018). Multiresidue determination of 21 pharmaceuticals in crayfish (*Procambarus clarkii*) using enzymatic microwave-assisted liquid extraction and ultrahigh-performance liquid chromatography-triple quadrupole mass spectrometry analysis. J. Pharm. Biomed..

[29] Madigosky S.R., Alvarez-Hernandez X., Glass J. (1991). Lead, cadmium, and aluminum accumulation in the red swamp crayfish *Procambarus clarkii* G. collected from roadside drainage ditches in Louisiana. Arch. Environ. Contam. Toxicol..

[30] Tkacikova S., Kozarova I., Mate D. (2010). Liquid chromatography tandem mass spectrometry determination of maduramycin residues in the tissues of broiler chickens. Food Addit. Contam. A.

[31] Nasz S., Debreczeni L., Rikker T., Eke Z. (2012). Development and validation of a liquid chromatographic-tandem mass spectrometric method for determination of eleven coccidiostats in milk. Food Chem..

[32] Chang K.C., Su J.J., Cheng C. (2013). Development of online sampling and matrix reduction technique coupled liquid chromatography/ion trap mass spectrometry for determination maduramicin in chicken meat. Food Chem..

[33] Hurst J.J., Wallace J.S., Aga D.S. (2018). Method development for the analysis of ionophore antimicrobials in dairy manure to assess removal within a membrane-based treatment system. Chemosphere.

[34] Dasenaki M.E., Thomaidis N.S. (2019). Multi-residue methodology for the determination of 16 coccidiostats in animal tissues and eggs by hydrophilic interaction liquid chromatography—Tandem mass spectrometry. Food Chem..

[35] Hu M., Wang Y., Yang J., Sun Y., Xing G., Deng R., Hu X., Zhang G. (2019). Competitive electrochemical immunosensor for maduramicin detection by multiple signal amplification strategy via hemin@Fe-MIL-88NH2/AuPt. Biosens. Bioelectron..

[36] Ni H., Peng L., Gao X., Ji H., Ma J., Li Y., Jiang S. (2019). Effects of maduramicin on adult zebrafish (*Danio rerio*): Acute toxicity, tissue damage and oxidative stress. Ecotoxicol. Environ. Saf..

[37] Ha J., Song G., Ai L.F., Li J.C. (2016). Determination of six polyether antibiotic residues in foods of animal origin by solid phase extraction combined with liquid chromatography-tandem mass spectrometry. J. Chromatogr. B.

[38] Barreca S., Busetto M., Vitelli M., Colzani L., Clerici L., Dellavedova P. (2018). Online solid-phase extraction LC-MS/MS: A rapid and valid method for the determination of perfluorinated compounds at sub ng.L-1 level in natural water. J. Chem..

[39] Dong H., Xian Y., Li H., Wu Y., Bai W., Zeng X. (2020). Analysis of heterocyclic aromatic amine profiles in Chinese traditional bacon and sausage based on ultrahigh-performance liquid chromatography-quadrupole-Orbitrap high-resolution mass spectrometry (UHPLC-Q-Orbitrap-HRMS). Food Chem..

