# Supplementary material for: Development and Validation of an Ultra-Performance Liquid Chromatography–Tandem Mass Spectrometry Method to Determine Maduramicin in Crayfish (Procambarus clarkii) and Evaluate Food Safety
Source: Foods. 2021 Feb 2;10(2):301. doi: 10.3390/foods10020301 (PMC7913001; doi:10.3390/foods10020301)
Supplement: Supplementary file 1 [file foods-10-00301-s001.pdf]

Supplemental data in *Foods*

Development and validation of an UPLC-MS/MS method for determination of maduramicin in crayfish (*Procambarus clarkii*) for food safety evaluation

Xiuge Gao[1,2], Pei Teng[1,2], Lin Peng[1,2], Hui Ji[1,2], Yawei Qiu[1,2], Xiaoxiao Liu[1,2], Dawei Guo[1,2]*, Shanxiang Jiang[1,2]*

[1]Joint International Research Laboratory of Animal Health and Food Safety, College of Veterinary Medicine, Nanjing Agricultural University, 1 Weigang, Nanjing 210095, PR China

[2]Laboratory of Veterinary Pharmacology and Toxicology, College of Veterinary Medicine, Nanjing Agricultural University, 1 Weigang, Nanjing 210095, PR China

* Dr. Dawei Guo and Dr. Shanxiang Jiang are co-corresponding authors.

Correspondence：

Dawei Guo, Ph.D., Associate Professor (gdawei0123@njau.edu.cn);

Shanxiang Jiang, Ph.D., Professor (nauvy@sina.com)

Joint International Research Laboratory of Animal Health and Food Safety,

Laboratory of Veterinary Pharmacology and Toxicology,

Nanjing Agricultural University,

1 Weigang, Nanjing 210095, PR China

Tel: 86-25-84396770

Fax: 86-25-84398669

Table S1. Mass spectrometric parameters of MAD and NIG.

Compound name	Qualitative ion (m/z)	Quantitative ion (m/z)	Cone voltage (V)	Collision energy (eV)
MAD	939.4>877.5	939.4>877.5	38	35
	939.4>896.3			40
NIG	742.7>657.6	742.7>657.6	50	30
	742.7>461.4			30

Note: MAD is short for maduramicin, NIG is short for nigericin, the same as below.

Table S2. Recovery rates of MAD in hepatopancreas and abdominal muscle of crayfish using three solid phase extraction columns (n=3).

Columns	Sample	Spiked concentration ($\mu g \cdot kg^{-1}$)	Mean recovery (%)	RSD (%)
Oasis HLB	Hepatopancreas	200	88.3	3.5
Strata C18		200	47.5	8.2
Sep-Pak Silica		200	52.6	6.4
Oasis HLB	Abdominal muscle	200	106.6	2.8
Strata C18		200	50.9	4.8
Sep-Pak Silica		200	55.4	7.5

Note: RSD is short for relative standard deviation.

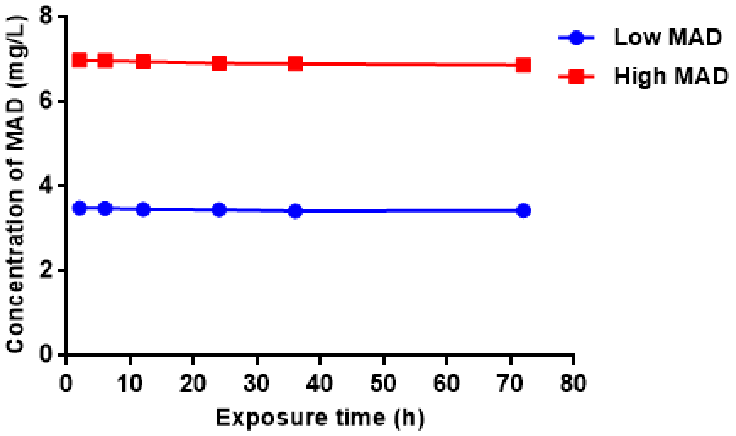

Figure S1. Concentration of MAD in water sample.
MAD in the water sample of exposure phase was determined using UPLC-MS/MS method. The fortified MAD was at 3.5 and 7 $mg \cdot L^{-1}$ as low and high exposure concentration.

Table S3. Standard solution curve and matrix-matched calibration curves of MAD.

Sample	Concentration range	Curve equation	r^2
Mobile phase	20–8,000 $\mu g \cdot L^{-1}$	y=0.0954x+0.5941	0.9974
Hepatopancreas	20–8,000 $\mu g \cdot kg^{-1}$	y=0.0980x+0.4991	0.9962
Abdominal muscle	20–8,000 $\mu g \cdot kg^{-1}$	y=0.0891x+0.6595	0.9977

Notes: y represents the MAD peak area / internal standard peak area and x represents concentration of MAD, r^2 represents the square of correlation coefficient of standard curve equation.

Table S4. Stability test results of MAD under three storage conditions (n=3).

Sample	MAD concentration / （$\mu g \cdot kg^{-1}$)	6 h RT stability		Freeze–thaw stability		30 d -80℃ stability	
		Mean recovery / (%)	RSD / (%)	Mean recovery / (%)	RSD / (%)	Mean recovery / (%)	RSD / (%)
Hepatopancreas	20	75.5	5.7	79.1	8.1	76.8	0.9
	200	94.1	5.9	89.3	2.2	92.8	0.8
	4000	90.2	1.6	89.7	1.2	90.5	1.4
Abdominal muscle	20	84.8	12.0	79.6	9.7	73.3	3.5
	200	108.4	0.6	108.8	1.1	106.0	0.9
	4000	99.3	1.0	102.6	2.5	101.2	1.1

Note: RT is short for room temperature.
